# Humoral and Cell-Mediated Responses to SARS-CoV-2 Vaccination in a Cohort of Immunodeficient Patients

**DOI:** 10.3390/hematolrep15040071

**Published:** 2023-12-08

**Authors:** Federica Plano, Mojtaba Shekarkar Azgomi, Anna Maria Corsale, Corinne Spoto, Nadia Caccamo, Serena Meraviglia, Francesco Dieli, Paolo D’Angelo, Antonino Trizzino, Sergio Siragusa

**Affiliations:** 1 Department of Health Promotion, Mother and Child Care, Internal Medicine and Medical Specialties, University of Palermo, 90127 Palermo, Italy; annamaria.corsale@unipa.it (A.M.C.); corinne.spoto@virgilio.it (C.S.); sergio.siragusa@unipa.it (S.S.); 2 Department of Biomedicine, Neurosciences and Advanced Diagnosis, University of Palermo, 90127 Palermo, Italy; mojtaba.shekarkarazgomi@unipa.it (M.S.A.); nadia.caccamo@unipa.it (N.C.); serena.meravigla@unipa.it (S.M.); francesco.dieli@unipa.it (F.D.); 3 Department of Pediatric Hemato-Oncology, ARNAS Ospedali Civico, G. Di Cristina, 90127 Palermo, Italy; oncoemato.ped@arnascivico.it (P.D.); antonino.trizzino@arnascivico.it (A.T.)

**Keywords:** COVID-19, vaccination, immunodeficiency, humoral-mediated response, cell-mediated response

## Abstract

This study delves into the intricate landscape of SARS-CoV-2 vaccine response in immunodeficient patients, focusing on the dynamics of both humoral and cell-mediated immunity. The cohort includes patients with common variable immunodeficiency (CVI), agammaglobulinemia (XLA), and combined immunodeficiency (CI). The findings reveal varying degrees of antibody production, with XLA patients exhibiting no measurable response but displaying a robust T-cell-mediated response. The study emphasizes the importance of considering both arms of the immune system in assessing vaccine immunogenicity, particularly in the context of immunodeficiency. The results challenge conventional measures of vaccine efficacy only based on antibody titers, highlighting the need for a more comprehensive understanding of the immune response in this vulnerable population. This research contributes valuable insights to guide clinical decisions regarding vaccination strategies, booster doses, and overall protection in immunodeficient individuals.

## 1. Introduction

The numerous clinical trials and the massive vaccination campaign against Coronavirus disease 2019 (COVID-19) worldwide have shown that the vaccine has excellent efficacy and a good safety profile, proving to be the best tool to prevent a severe form of the disease [1].

Immunodeficient patients are considered at high risk and have priority for vaccination because COVID-19 disease increases morbidity and mortality in these individuals [2].

Immunodeficiencies (ID) represent a class of heterogeneous diseases characterized by the impairment of distinct elements of the innate and adaptive immune system. They can be primary (PID), due to underlying genetic defects, or secondary (SID), due to hematologic malignancies (HM), immunosuppressive therapies, or hematopoietic stem cell transplantation (HSCT) [3].

IDs with predominant B lymphocyte impairment, such as Bruton’s agammaglobulinemia, X-linked agammaglobulinemia (XLA), or common variable immunodeficiency (CVID), and those with combined deficiencies, are characterized by a defect in antibody production ranging from the complete absence of antibodies to varying degrees of functional antibody abnormalities [4,5].

XLA is an inherited immunodeficiency disorder characterized by the absence of mature B cells [6], due to mutation of the BTK gene on the X chromosome that encodes for a signal-transducing tyrosine kinase that plays a role in the maturation of pro-B to pre-B cells [6]. Thus, in affected individuals, there is a failure of B-cell development resulting in immunoglobulin deficiency (hypogammaglobulinemia) or absence (agammaglobulinemia).

CVID is characterized by defects in the adaptive and innate immune system and, in particular, by abnormalities of B and T cells [7]. Most evidence shows that nearly 90% of patients with CVID have normal B cell numbers [8], indicating that the main defect is probably due to alterations in the terminal stages of B cell differentiation [9]. In fact, there is altered antibody production related to a defect in the differentiation of B cells into memory cells and plasma cells. In addition, several studies have reported decreased IgM memory B cells (CD19^+^CD27^+^), switched class memory B cells (CD19^+^CD27^+^IgD^−^IgM^−^), and plasma cells in patients with CVID [10,11].

T cell abnormalities have also been reported in patients with CVID, such as a reduction in the number of total CD4^+^ T cells and a relative increase in activated CD4^+^ T cells [12].

Because of the high risk of developing severe COVID-19, immunocompromised patients are considered a high priority for COVID-19 vaccination, as vaccination benefits are anticipated to outweigh the theoretical risks [13]. In fact, in individuals with PID, the infection–fatality ratio (IFR) was 20.0%, the case–fatality ratio (CFR) was 31.6%, and the inpatient mortality rate was 37.5%, while individuals with symptomatic SID had an IFR of 33.3%, a CFR of 39.2%, and an inpatient mortality rate of 44.0% [2,14].

mRNA vaccines are the vaccines that have been approved to date. Among these, mRNA-1273 by Moderna TX Inc and BNT162b1 by BioNTech-FosunPharma-Pfizer were the most used in Italy.

They consist of mRNA molecules encoding for the spike (S) protein, encapsulated in lipid nanoparticles (LNs) that facilitate mRNA penetration into the host cell [15]. The cellular immune response occurs when the S protein is presented to immune cells by the MHC class I or II complex, activating CD4^+^ and CD8^+^ T cells. However, the main mechanism of action is activating the humoral immune response. When naive B lymphocytes are activated by exposure to S proteins, they start to proliferate and differentiate into memory B cells or plasma cells with the ability to secrete antibodies [16].

A better understanding of the factors that regulate the response to vaccination in patients with PID or SID is needed to guide clinical decisions on the need for booster doses, the timing of vaccine administration, and general guidance on the level of protection achieved by vaccination.

Peripheral B lymphocytes are required for humoral vaccine response, which is often lower in these patients than in the normal population [17].

Experimental data suggest that T-cell responses (particularly CD8^+^ T cells) against SARS-CoV-2 infection may also play a protective role despite the presence of low or under-protective antibody levels [18]. Moreover, it has been shown that patients with agammaglobulinemia recover from COVID-19 in the absence of an antibody response, suggesting that T-cell responses may be sufficient to mediate protection or promote recovery from the disease [19]. In addition, several studies have shown that T-cell-mediated immune responses after COVID-19 vaccination have been detected in patients after anti-CD20 monoclonal antibody (mAb) rituximab treatment regardless of the presence or absence of B cells and antibody responses [17].

Several studies have already evaluated the antibody and the cell-mediated responses in a population of patients with PID two weeks after the second dose of the Pfizer-BioNTech vaccine. It has been shown that an effective T cell response is associated with less severe COVID-19 and this response is much more durable over time than the antibody-mediated response [16].

Therefore, both antibody- and cell-mediated responses should be considered when estimating vaccine immunogenicity. This is an even more important aspect in patients with immunodeficiency because, in most cases, there is a reduction in antibody production, so assessment of post-vaccine antibody titers alone may be reductive.

In this study, we have evaluated the S-specific antibody and T cell (CD4^+^ and CD8^+^)-mediated responses after anti-SARS-CoV-2 mRNA vaccination in a cohort of immunodeficient pediatric and adult patients with impairment of the B cell compartment because of PID or CD20-depleting therapy, to investigate how vaccination confers protection despite the deficiency of antibody response. In addition, we have correlated the laboratory data with the clinical characteristics of the different types of immunodeficiencies.

Finally, we have evaluated whether patients with PID or SID who receive the vaccine develop severe symptoms compared to the healthy vaccinated population.

## 2. Materials and Methods

### 2.1. Characteristics of Patients

The study was conducted in accordance with the Declaration of Helsinki, and approved by the AOUP Paolo Giaccone University Hospital Ethics Committee (protocol code 09/2021, October 2021).

Patients’ blood was drawn and analyzed between October 2021 and May 2022.

All blood samples were received from ARNAS Civico-Di Cristina-Benfratelli Hos-pital, Pediatric Oncohematology Unit, Palermo, Italy and processed in the AOUP Paolo Giaccone University Hospital, Central Laboratory of Advanced Diagnosis and Bio-medical Research (CLADIBIOR) Palermo Italy.

A population of 48 pediatric and adult patients with a mean age of 29 years was recruited in our study for the serological test, while only 30 patients were recruited for CD4 and CD8 T cell response to SARS-CoV-2 S protein. Four patients suffered from combined immunodeficiency, thirty-nine from common variable immunodeficiency, and five from agammaglobulinemia.

All of these patients have a form of primary immunodeficiency except for one who has immunodeficiency secondary to T-type lymphatic leukaemia and underwent chemotherapy and haploidentical marrow transplantation from his mother in 2013. Due to the characteristics of immunodeficiency, he was placed in the variable common type group by us. The main characteristics of patients are summarized (Table 1).

During the year prior to the study, in the cohort of patients an average rate of 2.2 infections was observed. Seventy percent of patients presented with one or more infections, of which the most frequent type was bronchitis. No infections required hospitalization. A total of 48% of patients presented with chronic sinus disease.

For serological analysis, samples from the patient population were compared with samples taken from a healthy population aged 35 to 62 years (mean age = 49) evaluated immediately after the second dose and after six months. Among the healthy subjects, there were 10 males and 11 females; the median age of the 21 subjects was 49 years.

Of the 48 patients recruited in the study, 26 contracted SARS-CoV-2 after the second dose of vaccine (between six and ten months later) with mild symptoms in 60% of cases and moderate symptoms in 40% of cases. Fifty percent of the healthy reference population had COVID-19, all with mild-to-moderate symptoms.

### 2.2. Sample Processing

First, serological testing with antibody assay was carried out in all patients recruited into the study, two months and six months after the second dose of mRNA vaccines.

The SPIKE anti-protein antibody (S1–S2) assay of SARS-CoV-2 was performed by Diasorin Liaison Sars CoV-2 Trimeric S-IgG assay.

The LIAISON^®^ SARS-CoV-2 TrimericSIgG is an indirect chemiluminescence immunoassay (CLIA) for the detection of IgG antibodies to SARS-CoV-2 in human serum and plasma samples. The principal components of the test are magnetic particles (solid phase) coated with recombinant trimeric SARS-CoV-2 spike protein and a conjugate reagent containing an anti-human IgG mouse monoclonal antibody linked to an isoluminol derivative (isoluminol-antibody conjugate).

Lymphocyte subpopulation assessment tests were additionally performed in peripheral blood. Subpopulation analysis on peripheral blood was conducted by multiparameter flow cytometry on nucleated material obtained after lysis of erythrocyte populations by lyse/no wash method. Leukocyte distribution (CD45/SSC) and T, B populations were evaluated by assessing their percentage and absolute counts. Monoclonal antibodies used were: CD45, CD3, CD16, CD56, CD4, CD8, CD19, CD20, CD14, anti-HLA-DR, anti-kappa, anti-Lambda, anti-TCRαβ, anti-TCR-γδ, CD38, CD24, CD27, CD183, CD196, CD25, and CD5.

The absolute number (*n* cells ×10^3^/µL) of naive and B memory B lymphocytes were obtained by calculating their percentage from the absolute number of CD19^+^ CD20^+^ lymphocytes.

In addition, the cell-mediated response to the vaccine was assessed by a T lymphocyte stimulation test with the S protein of SARS-CoV-2.

This assay is based on the detection of the production of three cytokines (IL-2, IFN-γ, and TNF-α) by T cells after in vitro stimulation with a pool of lyophilized peptides, consisting mainly of 15-mer sequences with an overlap of 11 amino acids, covering the immunodominant sequence domains of the spike sequence domains of the SARS-CoV-2 S glycoprotein (PepTivator^®^ SARS-CoV-2 Prot._S, Miltenyi Biotec, Surrey, UK).

PBMCs were stimulated for 18 h at 37 °C 5% CO_2_ in RPMI1640 complete medium with a pool of spike-specific peptides (1 µg/mL) at 1 × 10^6^ cells/mL. RPMI or ionomycin/PMA were included in each sample as negative or positive controls, respectively. Brefeldin-A (10 µg/mL) was added to the cells after 2 h of incubation to force them to retain cytokines.

After 18 h of stimulation, cells were collected and stained, first with live/dead marker (Zombie dye, Biolegend, San Diego, CA, USA) and then with mAb anti-human CD3PerCP-Vio700, mAb anti-human CD4 PE-Vio^®^ 770, and mAb anti-human CD8 APC. After surface staining, cells were fixed, permeabilized, and stained at room temperature for 30 min with mAb to anti-human IL-2 APC-Vio^®^ 770, mAb anti-human IFN-γ FITC, and mAb anti-human TNF-α PE. Samples were acquired on a FACSARIA II flow cytometer (BD Bioscience, San Jose, CA, USA) and analyzed using FlowJo v10 (BD Bioscience, San Jose, CA, USA).

The threshold for positivity for S-specific CD4^+^ T cell responses (>0.02%) and S-specific CD8^+^ T cell responses (>0.05%) was set according to Dan et al. [20] and calculated using the median two-fold standard deviation of all negative controls measured. GraphPad software was used to perform statistical analysis.

## 3. Results

### 3.1. Antibody Response: Anti-SARS-CoV-2 IgG Value Evaluated Immediately after the Second Dose and after Six Months and Compared to Healthy Subjects

ID patients were tested for antibody response two and six months after the second dose of the vaccine.

**Figure 1:**
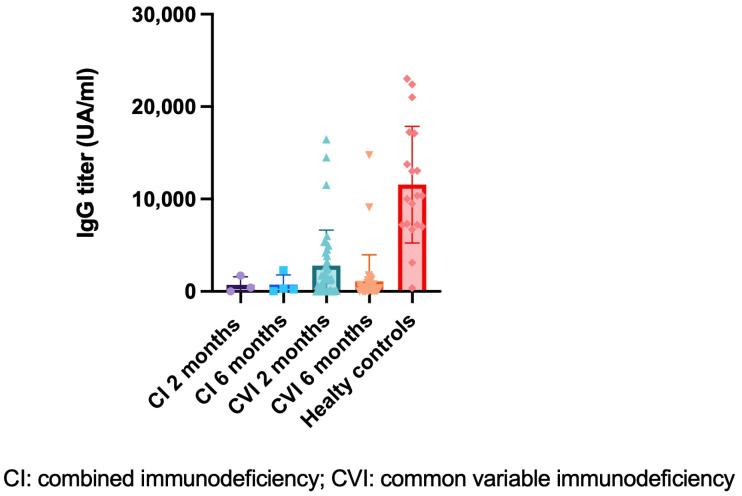
Comparison of specific anti-recombinant trimeric SARS-CoV-2 spike protein IgG titers two and six months after second vaccine dose between healthy subjects and immunodeficient patients (combined immunodeficiency and common variable immunodeficiency).

Comparing to age- and sex-matched healthy controls, the antibody response in patients with immunodeficiency (combined immunodeficiency and common variable immunodeficiency) at two months after the second dose of the vaccine was consistently lower (mean IgG in patients = 3137 UA/mL vs. healthy controls = 14,268, Figure 1).

However, the differences were not statistically significant (*p* = 0.446).

Patients with agammaglobulinemia did not produce S-specific antibodies, and titers were below 0 at any time after vaccination.

Patients with combined immunodeficiency and patients with variable common immunodeficiency both had good S-specific IgG production after the second dose of the vaccine, with titers >15.0 UA/mL (this is the cut-off for positivity). In all cases, however, the antibody titers at six months were 50% lower than titers at two months.

There were no statistically significant differences between antibody responses in patients with variable common immunodeficiency and those with combined immunodeficiency at two months (mean IgG = 2781.74 UA/mL vs. 707 UA/mL, *p* = 0.351) and at six months (mean IgG = 1094 UA/mL vs. 725 UA/mL, *p* = 0.614).

### 3.2. T-Cell-Mediated Response: Comparison of CD4 and CD8 T Cell Responses and Cytokine Production Assay to SARS-CoV-2 Protein S

Next, we assessed the CD4 and CD8 T-cell-mediated responses to a pool of peptides derived from the S protein of SARS-CoV-2 by intracellular cytokine staining (ICS). Because of limited cell numbers, only 30 out of the 49 patients were tested. A quantitative assessment of S-specific CD4 and CD8 T cells was performed and expressed as the number of responder cells/10^6^ cells (after subtracting negative control values).

**Figure 2:**
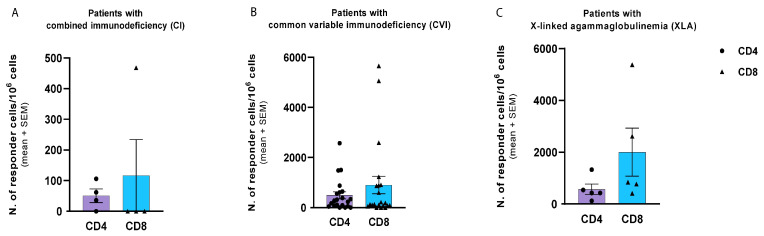
Comparison of CD4 and CD8 T cell responses to SARS-CoV-2 S protein. Patients with combined immunodeficiency (**A**), with common variable immunodeficiency (**B**), and with agammaglobulinemia (**C**). Number (*N*) of patients with CI: 4; *N*. of patients with CVI: 21-, *N*. of patients with XLA: 5.

As shown in Figure 2, the CD4 T-lymphocyte-mediated response is lower than the CD8 response in all groups of patients with immunodeficiency, but differences are not statistically significant (*p* = 0.070). For patients with combined immunodeficiency, the mean was 51 × 10^6^ cells for CD4 response and 117 × 10^6^ cells for CD8 response.

The S-specific CD4 and CD8 T cell responses were almost absent in patients with variable common immunodeficiency (mean 328 × 10^6^ cells and 315 × 10^6^ cells, respectively) but above the reference cut-off (positive reference value > 200 × 10^6^ cells for CD4 response and >500 × 10^6^ cells for CD8 response [21]) in patients with agammaglobulinemia (mean 568 × 10^6^ cells for CD4 response and 2004 × 10^6^ cells for CD8 response).

We then analyzed qualitatively the CD4 and CD8 T cell responses, by measuring distinct intracellular cytokines upon S-derived peptide stimulation.

**Figure 3:**
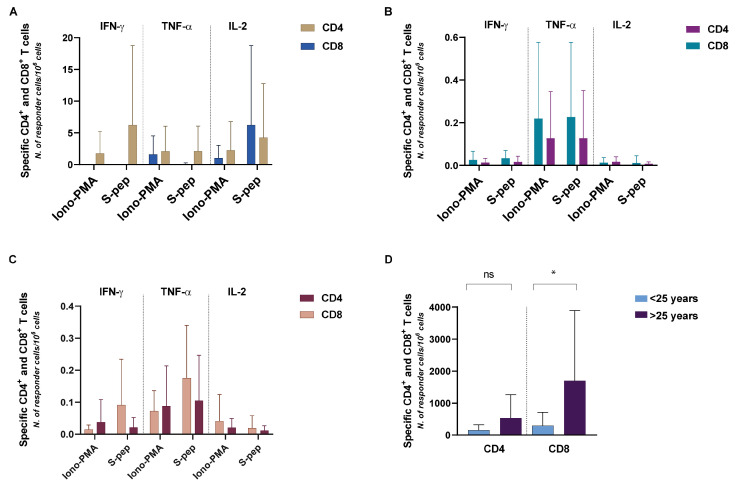
Analysis of CD4^+^ and CD8^+^ T cells cytokine expression in response to S-derived peptides in patients with combined immunodeficiency (**A**), with common variable immunodeficiency (**B**), and with agammaglobulinemia (**C**); comparison of CD4^+^ and CD8^+^ T cell responses in patients <25 years old and those >25 years (**D**). The histograms show mean plus SD. ns = not statistically significant; * = *p* value < 0.05. *N*. of patients with CI: 4; *N*. of patients with CVI: 21-, *N*. of patients with XLA: 5.

In patients with combined immunodeficiency, the CD4 lymphocyte response was characterized by both IFN-γ and IL-2 production (Figure 3A).

CD8 T cells only expressed IL-2, but neither IFN-γ nor TNFα (Figure 3A).

In patients with common variable immunodeficiency (Figure 3B), there was a very poor number, if any, of S-specific CD4^+^ and CD8^+^ T cells expressing cytokines (IFN-γ, IL-2, and TNF-α).

In patients with agammaglobulinemia (Figure 3C), there was no S-specific cytokine expression by CD4 T lymphocytes, but consistent IFN-γ and TNF-α expression by CD8^+^ T cells.

Finally, we correlated CD4 and CD8 T-cell-mediated responses with age, after clustering patients in two groups: <25 years (young) and >25 years (adult). We found that young patients show a quantitatively lower T-cell-mediated response than adult patients, which is evident for both CD4 (*p* = 0.473) and CD8 responses (*p* = 0.032), although only the latter attains statistical significance (Figure 3D).

**Figure 4:**
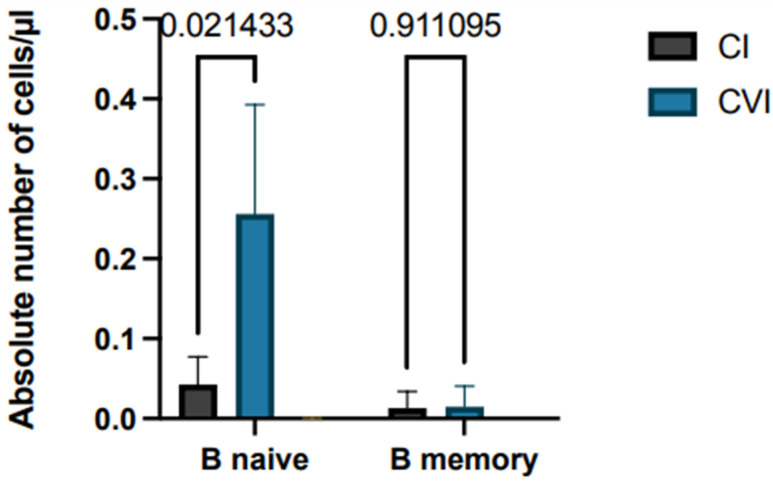
Analysis of lymphocyte subpopulations (B memory and B naive) of immunodeficiency patient subgroups; the histograms show mean plus SD. *N*. of patients with CI: 4; *N*. of patients with CVI: 21-.

Finally, lymphocyte subpopulations in patients with immunodeficiency were correlated with response to vaccination for SARS-CoV-2, as differences between naive and memory B lymphocytes may impact on antibody production. Patients with combined immunodeficiency have similar numbers of these two B cell subpopulations: mean 0.014 B naive/µL vs. 0.013 B memory/µL. In the case of patients with variable common immunodeficiency, naive B cell numbers were greater than B memory cell numbers (mean 0.256 B naive/µL vs. 0.015 B memory/µL) with statistically significant difference (*p* = 0.02), while patients with agammaglobulinemia showed absence of B cells or the B memory cell subset (Figure 4).

## 4. Discussion

Immunization is the most effective intervention for the prevention of infectious diseases. Vaccination against SARS-CoV-2 has proven to be extremely useful and safe, limiting viral spread and preventing the severe form of the disease. However, data on the efficacy of the anti-SARS-CoV-2 vaccine in individuals with primary and secondary immunodeficiency are still preliminary and studies are needed to encourage such patients to undergo vaccination [21].

Numerous studies have also shown that mRNA vaccines induce a reduced humoral response in patients with primary or acquired immunodeficiencies [22], but stimulate a cell-mediated immune response [23,24]. Therefore, it would be desirable to estimate the efficacy of vaccination by also measuring T-cell-mediated immune response.

In addition, hematologic malignancy has been widely shown to be an independent predictor of COVID-19 mortality after adjustment for Eastern Cooperative Oncology Group (ECOG) performance and disease status [25].

In this study, we stressed the importance of cell-mediated immunity to SARS-CoV-2 vaccine mRNA to encourage even immunodeficient patients to receive a vaccination, regardless of the humoral response.

We first show here that (and as expected) patients with XLA have no antibody production after the two doses of the vaccine; nevertheless, none of them developed the severe form of the disease after becoming infected with SARS-CoV-2.

In these patients, T-cell-mediated response is higher than in those with CVI and CI, with absent cytokine expression by CD4 T lymphocyte and expression of IFN-γ and TNF-α by CD8 T lymphocytes. Also, it is noteworthy that these patients have the lower number of naive B cells among all three groups, as the B lymphocytes fail to differentiate into memory B cells.

In patients with CI, the antibody response was lower than those with CVI, the CD4^+^ T lymphocyte response was characterized by IFN-γ and IL-2 production and the CD8 response by IL-2 production.

Of note, two patients (one with CI and one with XLA) showed the absence of both antibody- and cell-mediated responses. One of the two patients had COVID-19 in mild/moderate form and after recovery he developed an antibody response with IgG = 1300 UA/mL.

In these patients, defects are present not only in B lymphocytes but also in other immune cells involved in generating an effective humoral response, including antigen-presenting cells and Th cells. Th1 cells produce IFN-γ and TNF-α, which stimulate macrophage activation and cell-mediated immunity, while Th2 cells produce IL-4 and IL-13, which promote B-cell maturation and antibody production [26]. Impaired Th cells function in patients with CI limits T–B cooperation, exacerbating the B cell defect. However, the CD4^+^ T cell response after stimulation with S protein underscores how the vaccine elicits a cell-mediated response, likely to protect against the virus. In fact, only two patients with CVI contracted SARS-CoV-2 with almost no symptoms.

Finally, one of the criteria for the diagnosis of CVI is poor antibody response to vaccines. Neoantigen challenge antibody response testing is used to assess antibody production in patients who are already receiving immunoglobulin replacement therapy. Therefore, assessing the humoral response to SARS-CoV-2 in vaccinated patients could have been another neoantigen challenge. However, the high percentage of immunodeficiency patients who responded to the vaccine, including patients diagnosed with CVI, suggests that vaccine response should be interpreted with caution and that a positive antibody response does not exclude a clinically significant antibody deficiency [27].

This study makes us realize how important vaccination is in immunocompromised patients and that serologic study alone cannot be used as a parameter to measure vaccine-induced responses.

In conclusion, we demonstrate and emphasize here the importance of anti-SARS-CoV-2 vaccination in patients with immunodeficiency.

Through a simple test, we have shown how it is possible to detect and quantify vaccine-induced specific CD4^+^ and CD8^+^ T cells, thus monitoring the response to the vaccine. This is useful because it shows that even in the absence of a humoral response, the T cell response is often preserved.

Our study shows that vaccination with mRNA vaccines induces a specific T cell-mediated immune response against SARS-CoV-2 independently of the B cell response.

## 5. Conclusions

Through a simple test, we have shown how it is possible to detect and quantify vaccine-induced specific CD4^+^ and CD8^+^ T cells, thus monitoring the response to the vaccine.

This article explores the response to SARS-CoV-2 vaccination in immunodeficient patients, particularly those with common variable immunodeficiency (CVI), agammaglobulinemia (XLA), and combined immunodeficiency (CI). The study focuses on both humoral and cell-mediated immunity, challenging the conventional assessment of vaccine efficacy based solely on antibody titers. Our study shows that vaccination with mRNA vaccines induces a specific T-cell-mediated immune response against SARS-CoV-2 independently of the B cell response.

The findings reveal varying degrees of antibody production, with XLA patients showing no measurable response but exhibiting a robust T-cell-mediated response. This is useful because it shows that even in the absence of a humoral response, the T cell response is often preserved. The research emphasizes the need for a comprehensive understanding of the immune response in immunodeficient individuals and suggests that both antibody- and cell-mediated responses should be considered in assessing vaccine immunogenicity. The study also highlights the importance of vaccination in immunocompromised patients, even in the absence of a robust humoral response. The results contribute valuable insights to guide clinical decisions regarding vaccination strategies, booster doses, and overall protection in this vulnerable population.

In summary, the article underscores the significance of evaluating both arms of the immune system in immunodeficient patients after SARS-CoV-2 vaccination and emphasizes the role of T-cell-mediated responses in providing protection.

## Figures and Tables

**Table 1:** The main characteristics of patients enrolled in the study.

	Number	Rate (%)	Mean	Range
Total	48	100		
Age			29	6–65
Sex				
Female	21	44		
Male	27	56		
Immunodeficiency				
Combined immunodeficiency	4	9		
Common variable immunodeficiency	39	81		
Agammaglobulinemia	5	10		
SarsCov2 infection after vaccine	26	54		
Symptoms during SarsCov2 infection				
Severe	0	0		
Moderate	3	12		
Mild/asymptomatic	23	88		

## Data Availability

Data are contained within the article.
